# Combination of 3D chromatin architecture and omics analysis provides insight into anthocyanin regulation in *Actinidia arguta*

**DOI:** 10.1093/hr/uhaf183

**Published:** 2025-07-22

**Authors:** Yukuo Li, Zhe Song, Xu Zhan, Xiaohan Li, Lingshuai Ye, Miaomiao Lin, Ran Wang, Leiming Sun, Hong Gu, Jinyong Chen, Jinbao Fang, Xiujuan Qi

**Affiliations:** National Key Laboratory for Germplasm Innovation & Utilization of Horticultural Crops, Zhengzhou Fruit Research Institute, Chinese Academy of Agricultural Sciences, Zhengzhou 450009, China; Zhongyuan Research Center, Chinese Academy of Agricultural Sciences, Xinxiang 453519, China; National Key Laboratory for Germplasm Innovation & Utilization of Horticultural Crops, Zhengzhou Fruit Research Institute, Chinese Academy of Agricultural Sciences, Zhengzhou 450009, China; School of Agricultural Sciences, Zhengzhou University, Zhengzhou 450001, China; National Key Laboratory for Germplasm Innovation & Utilization of Horticultural Crops, Zhengzhou Fruit Research Institute, Chinese Academy of Agricultural Sciences, Zhengzhou 450009, China; College of Horticulture and Plant Protection, Henan University of Science and Technology, Luoyang 471000, China; National Key Laboratory for Germplasm Innovation & Utilization of Horticultural Crops, Zhengzhou Fruit Research Institute, Chinese Academy of Agricultural Sciences, Zhengzhou 450009, China; School of Agricultural Sciences, Zhengzhou University, Zhengzhou 450001, China; National Key Laboratory for Germplasm Innovation & Utilization of Horticultural Crops, Zhengzhou Fruit Research Institute, Chinese Academy of Agricultural Sciences, Zhengzhou 450009, China; National Key Laboratory for Germplasm Innovation & Utilization of Horticultural Crops, Zhengzhou Fruit Research Institute, Chinese Academy of Agricultural Sciences, Zhengzhou 450009, China; Zhongyuan Research Center, Chinese Academy of Agricultural Sciences, Xinxiang 453519, China; National Key Laboratory for Germplasm Innovation & Utilization of Horticultural Crops, Zhengzhou Fruit Research Institute, Chinese Academy of Agricultural Sciences, Zhengzhou 450009, China; National Key Laboratory for Germplasm Innovation & Utilization of Horticultural Crops, Zhengzhou Fruit Research Institute, Chinese Academy of Agricultural Sciences, Zhengzhou 450009, China; National Key Laboratory for Germplasm Innovation & Utilization of Horticultural Crops, Zhengzhou Fruit Research Institute, Chinese Academy of Agricultural Sciences, Zhengzhou 450009, China; National Key Laboratory for Germplasm Innovation & Utilization of Horticultural Crops, Zhengzhou Fruit Research Institute, Chinese Academy of Agricultural Sciences, Zhengzhou 450009, China; National Key Laboratory for Germplasm Innovation & Utilization of Horticultural Crops, Zhengzhou Fruit Research Institute, Chinese Academy of Agricultural Sciences, Zhengzhou 450009, China; National Key Laboratory for Germplasm Innovation & Utilization of Horticultural Crops, Zhengzhou Fruit Research Institute, Chinese Academy of Agricultural Sciences, Zhengzhou 450009, China; Zhongyuan Research Center, Chinese Academy of Agricultural Sciences, Xinxiang 453519, China

## Abstract

*Actinidia arguta* has become popular with consumers recently because of its edible and colorful fruit skin. The 3D spatial organization of its genome plays a key role in the formation of various biological traits. However, the function of 3D genome reorganization during fruit skin color formation is poorly understood in *A. arguta*. In this study we constructed the 3D genome of the red-skinned *A. arguta* cultivar ‘Zhonghongbei’ (ZHB) and the green-skinned cultivar ‘Zhonglvbei’ (ZLB), and performed chromatin structure comparisons between them at compartment, topologically associating domain (TAD), and loop levels. Global compartment comparisons at whole 3D genome level between red-skinned and green-skinned *A. arguta* showed that A–B compartment transition specifically occurred in chromosome 7 and chromosome 16, based on which all genes within 3 Mb upstream and downstream of A–B compartment transition were retrieved to construct a four-way Venn diagram, which showed that *AaCBP60B-like*, encoding calmodulin-binding protein 60 B-like, is the key candidate gene negatively correlating with fruit color. Exogenous calcium chloride treatments enhancing *AaCBP60B-like* expression to repress anthocyanin biosynthesis proved a negative role of *AaCBP60B-like* in anthocyanin biosynthesis. Overexpression and virus-induced gene silencing assays of *AaCBP60B-like* revealed the inhibition of anthocyanin biosynthesis derived from differential expression of *AaCBP60B-like* resulting from a 346-bp InDel variation located at the *AaCBP60B-like* promoter resulting in activity differences in red- and green-skinned *A. arguta*. ATAC-seq results proved that the 346-bp InDel variation affects 3D genome organization. Our study provides the first 3D chromosome organization in red- and green-skinned *A. arguta*, based on which a candidate gene, *AaCBP60B-like*, involved in anthocyanin regulation is identified.

## Introduction

*Actinidia arguta* is a new kind of *Actinidia* species that has been commercially cultivated in recent years, and it has special fruit traits, including mini fruit size with average weight of just ~20 g, edible fruit skin that is easy to eat without peeling, differing from the traditional big-fruit type, *Actinidia chinensis* [1]. The attractive skin appearance is of great importance for marketing. The skin can mainly be divided into red and green types; the former gradually accumulates red pigments resulting from anthocyanin biosynthesis during fruit ripening, whereas the latter stays green during the whole of fruit development. Red-skinned *A. arguta* generally has a higher price because of its red appearance and richness in anthocyanin. Anthocyanin biosynthesis and regulation have been well studied in *Actinidia*. MYB/bHLH/WD40-formed MBW protein complex is involved in anthocyanin regulation. AcMYB10 and AcMYB110 delicately regulated anthocyanin biosynthesis in inner pericarp and whole flesh of *A. chinensis*, respectively [2]. In our previous study, we demonstrated that AaMYBC1 participates in anthocyanin regulation by interacting with AabHLH42 to form MYB/bHLH complexes in *A. arguta* flesh [3]. Subsequently, AaMYBC1 was found to have a new proanthocyanin-related function in regulating branch points in the anthocyanin pathway, together with AaWRKY44 [4]. Besides, transcription factors including MYB75, MYBF110, MYB123, and MADS68 were also proved to be involved in anthocyanin regulation [5–8]. On the one hand, these are mostly identified as positive regulators of anthocyanin biosynthesis, which are rarely used as the target for gene editing in the future for new red skin germplasm creation; on the other hand, most of these regulators were identified in traditional ways, such as reverse genetic approaches, that focus on gene expression or potential function rather than the whole landscape regulation of fruit coloration, which limits our comprehensive understanding of the mechanism of anthocyanin regulation. New technology used for mining new negative regulators and exploring the mechanism of anthocyanin regulation in *A. arguta* skin is awaited.

High-throughput chromosome conformation capture (Hi-C), as a useful tool for 3D genome analysis, permits us to explore the organization of chromatin and its role in gene expression and regulation, controlling various biological processes in eukaryotic organisms [9–14]. 3D chromatin organization maps have already been reported in plants including rice [15], maize [16], soybean [17], tea [18], *Brassica* crops [19] and barley [20]. 3D genome architecture characterization of diploid and tetraploid cotton provided novel light on the relationship between 3D genome evolution and transcription regulation by comparison between each of the sub-genomes at different levels of 3D organization, including A/B compartments and topologically associated domains (TADs) [21]. Additionally, chromatin interaction maps for *Brassica rapa* and *B. oleracea* revealed the role of 3D organization in genome evolution in the *Brassica* genus [22]. Hi-C was also used for studying transcription regulation in response to environmental change. Knowledge of features such as higher-order chromatin architecture could provide a new means of understanding the role of reorganization of 3D chromatin architecture in the regulation of gene expression during heat stress in rice [23]. 3D chromatin architecture capture technology is a useful tool for biological phenotype clarification, giving new insight in plants, but it is little used in kiwifruit.

With the fruit development and ripening process, red and green skin of *A. arguta* presents significant color variation, yet the underlying mechanism of chromatin organization involved in the color regulation remains largely unclear. Based on our previous chromosome-level genome assembly of the red *A. arguta* cultivar ‘Tianyuanhong’ [24], in this study we conducted Hi-C experiments between red- and green-skinned *A. arguta*. The expected chromatin transitions were observed through the whole genome. We combined comparative genome, RNA-seq, and ATAC-seq results with Hi-C differences to show the comprehensive landscape, and screened *AaCBP60B-like* as a key negative regulator in fruit skin color of *A. arguta*, and showed that the 346-bp InDel variation in the *AaCBP60B-like* promoter can be used as the target design for breeding by gene editing.

**Figure 1:**
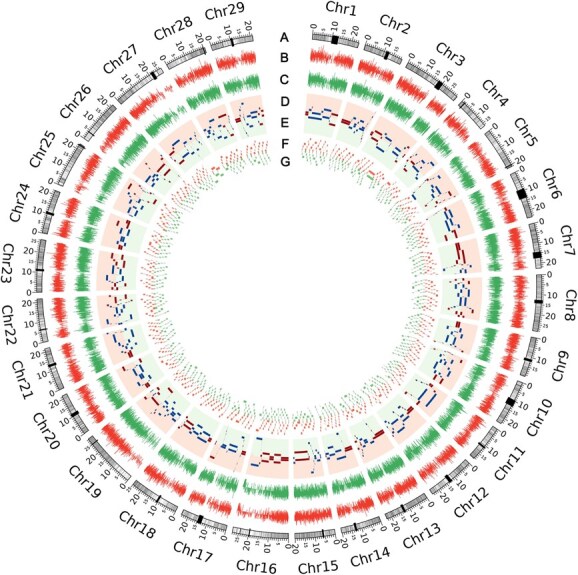
Circos plots of chromatin 3D structure comparison between ZHB and ZLB *A. arguta*. Eight rings from outside to inside show: A, genomic positions; black blocks located at chromosomes represent centromeres. B and C, RNA FPKM (fragments per kilobase per million mapped reads) of gene expression for ZHB/ZLB skin. D and E, A/B compartment for ZHB/ZLB skin, different color in track for A and B compartments, respectively. F and G, TAD separation score for ZHB/ZLB skin.

**Figure 2:**
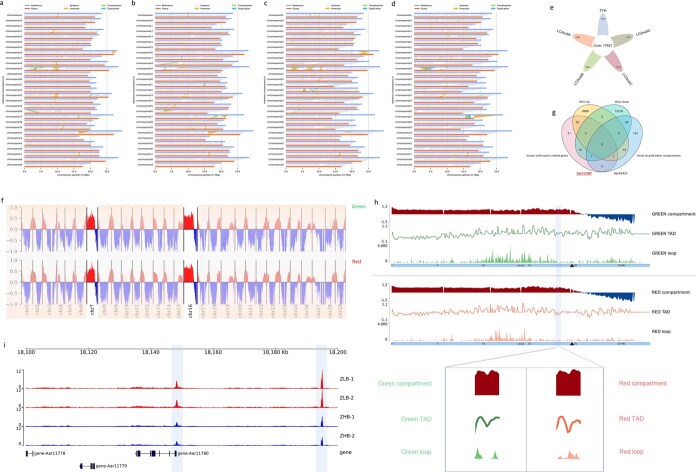
Combination analysis to screen the key candidate gene. (a–d) Structural variation analysis through the whole genome between TYH and four LC2 sub-genomes. The structural variations identified between TYH and LC2 subA–D, including syntenic regions, translocations, inversions, and duplications, were visualized using color-coded blocks. (e) Statistics of core genes and dispensable genes of TYH and LC2. (f) Global compartment bar plot at whole-genome level between ZHB and ZLB *A. arguta*. Chromosomes 7 and 16 exhibit significantly different compartment A/B distribution patterns relative to other chromosomes. (g) Four-way Venn diagram to directly screen candidate Aar11780. The four gene categories included: upregulated DEGs, downregulated DEGs, known anthocyanin-related genes, and genes located within the compartments. These were used for direct target screening. (h) Chromatin structure analysis of *Aar11780* in chromosome 16. The differences between ZHB and ZLB *A. arguta* at three levels, including compartment, TAD, and loop, are magnified to shown them clearly. The horizontal axis shows the genomic position on chromosome 16, while the vertical axis displays the bin values for three chromatin features: compartments, TADs, and loops. (i) Specific peak comparison of Aar11780 in chromosome 16 between ZHB and ZLB from ATAC-seq analysis. The horizontal axis shows the specific gene position on the chromosome, while the vertical axis displays the accessibility score of chromatin for the peak plot. Higher peak values (signal intensity) indicate greater chromatin accessibility.

## Results

### Chromatin 3D structure comparison between red- and green- skinned *A. arguta*

Reproducibility of replicate experiments was assessed by measuring concordance of contact maps using GenomeDISCO [25] to ensure Hi-C analysis accuracy. There was high reproducibility of the Hi-C experiments based on the high correlation of chromatin interactions in our experiments (Supplementary Data Fig. S1, Supplementary Data Tables S1 and S2). Normalized Hi-C matrices were constructed for principal component analysis (PCA) to investigate long-range compartmentalization, including A compartments generally, as open chromatin was more related to euchromatic, gene enrichment, and high transcription activity regions, and B compartment, as closed chromatin, was more related to heterochromatin, gene non-enrichment, and low transcription activity regions [26]. Notably, more genes were located in A compartments in red-skinned *A. arguta* ZHB than in green-skinned *A. arguta* ZLB (Supplementary Data Table S3), suggesting more potential genes were expressed in ZHB during skin coloration. We further analyzed local-level chromatin organization (TADs) and whole-genome *cis*-interaction (Supplementary Data Table S4, Supplementary Data Fig. S2), and found conserved chromatin interaction between red- and green-skinned *A. arguta*. To intuitively exhibit differences in chromatin 3D structure of ZHB versus ZLB, we compared compartment A/B, TADs, and loops, coupled with gene expression based on RNA-seq data that have been submitted to the public database Genome Sequence Archive (GSA) with the accession number CRA020742. Of note, compartment B dominated in most chromosomes, except for chromosome 7 and chromosome 16, in which compartment A dominated (Figs 1 and 2a), indicating more active genes highly expressed in chromosome 7 and chromosome 16. We identified 5895 stable (A2A and B2B) and 344 switching (A2B and B2A) compartments (Supplementary Data Fig. S3, Supplementary Data Table S5), and found the B compartments in red-skinned *A. arguta* converted to A compartments in green-skinned *A. arguta* both in chromosome 7 and chromosome 16 (Supplementary Data Fig. S4), indicating some silencing anthocyanin-related repressors in red-skinned *A. arguta*, while there might be active status in green-skinned *A. arguta* in chromosome 7 and chromosome 16. In particular, chromosome 16 contains some anthocyanin-related genes such as *F3GT* (*Aar11639*) and three *F3H* genes (*Aar10772*, *Aar10906*, *Aar11322*) (Supplementary Data Table S6), indicating chromosome 16 might have functional regulators involved in anthocyanin biosynthesis.

### Screening of candidate genes using multi-omics analysis

Previously, we have assembled the chromosome-level high-quality genome of the red autotetraploid *A. arguta* cultivar ‘Tianyuanhong’ (TYH) [24], based on which we conducted a comparative genome analysis using TYH as the query genome to map the green autotetraploid *A. arguta* LC2 with released genome [27] to identify the structural variations at the whole-genome level, which showed that presence and absence variation (PAV) is the major contributor to the genome variations. Inversions and translocations are relatively less frequent than copy number variations (CNVs) and PAVs in comparisons between the TYH and LC2 genomes. Notably, a higher numbers of CNVs and PAVs are distributed in TYH vs LC2subA and TYH vs LC2subB compared with TYH vs LC2subC and TYH vs LC2subD. This suggests that allele-specific CNVs or PAVs may contribute to the functional differences related to fruit skin color (Table 1, Fig. 2b–e). There was a total of 17 621 core genes between TYH and LC2, and 13 795 dispensable genes in TYH compared with LC2 (Fig. 2f). To further identify the candidate genes involved in anthocyanin regulation, we retrieved the genes located in the 3-Mb region in the upstream and downstream regions of compartment transition both in chromosome 7 (Supplementary Data Fig. S5) and chromosome 16 (Supplementary Data Fig. S6), in which a total of 249 genes were obtained (Supplementary Data Table S7). To identify the early, mid, and late-phase gene expression patterns, as well as transient regulatory changes that are closely associated with phenotypic alterations in fruit color, we carried out a time-course differentially expressed gene (DEG) analysis comparing the red-skinned cultivar ZHB with the green-skinned cultivar ZLB, particularly during the three key developmental stages (S1, S2, and S3) of fruit coloration. A total of 8673 up-regulated genes and 12 297 down-regulated genes were identified in the three comparisons including ZHB vs ZLB_S1, ZHB vs ZLB_S2 and ZHB vs ZLB_S3 (Supplementary Data Fig. S7, Supplementary Data Table S8). Next, we conducted a more robust bioinformatics approach to construct a four-way Venn diagram that intersected (i) known anthocyanin-related genes, (ii) the up-regulated DEGs-Up, (iii) the down-regulated DEGs obtained from the comparisons, and (iv) genes located within compartments that are implicated in chromatin reorganization. Two candidates, *Aar41421* and *Aar11780*, were immediately screened to have positive and negative correlation with fruit skin color (Fig. 2g, Supplementary Data Table S9). *Aar41421* is annotated as *F3GT*, which is the known structural gene involved in the anthocyanin biosynthesis pathway. We focused on the candidate gene *Aar11780*.

To confirm whether *Aar11780* is the candidate gene, we also conducted weighted gene coexpression network analysis (WGCNA) using the R package. We set soft-thresholding power as 7 (scale-free *R*^2^ > 0.8) to establish adjacency matrix (Supplementary Data Fig. S8a and b), and identified six modules, in which correlations above 0.75 were merged together (Supplementary Data Fig. S8c and d). Next, we determined correlations between modules and color traits to understand the biological significance of these modules. Most anthocyanin-related genes previously identified are mainly positive regulators; here, what we want to mine are negative genes involved in anthocyanin regulation. So we focused on the negative correlation in the heat map of module colors. We identified that the MEturquoise module was most negatively correlated with Red-S1 traits, which is the unique green sample compared with Red-S2 and Red-S3 (Supplementary Data Fig. S8e). By setting module membership (MM) higher than 0.8, we eventually selected 42 hub genes from the MEturquoise module (Supplementary Data Table S10). The expression level of these 42 hub genes in Red-S1, Red-S2, and Red-S3 samples showed that six candidate genes, *Aar11780*, *Aar41407*, *Aar41224*, *Aar41362*, *Aar41299*, and *Aar41302*, differentially clustered with other genes (Supplementary Data Fig. S9). The functional annotations of these six candidates indicated that *Aar11780* is the calmodulin-binding protein 60 B-like gene that has been reported to be involved in anthocyanin anthocyanin in *Arabidopsis* [28] (Supplementary Data Table S11), so we deduced that *Aar11780* might be the key candidate gene in kiwifruit anthocyanin regulation. Furthermore, the 3D-level analysis showed that *Aar11780* belonged to compartment A both in red-skinned *A. arguta* ZHB and green-skinned *A. arguta* ZLB, but the bin value differed significantly in ZHB and ZLB (Fig. 2h). The TAD quantitative metrics were −0.0888902 in ZLB and −0.429176 in ZHB, while the loop values were 0 in ZLB and 61 in ZHB (Fig. 2h, Supplementary Data Table S12), suggesting TAD and loop level in ZLB varied more than in ZHB as well, and indicating that *Aar11780* is the gene affected by 3D genome level. Furthermore, we conducted ATAC-seq analysis, which showed higher peak enrichment at the *Aar11780* position in ZLB than in ZHB, implying *Aar11780* has higher activity at 3D chromatin level in ZLB (Fig. 2i). Notably, compartment A/B occurred transition nearby, downstream of *Aar11780* at chromosome 16, as described in the Hi-C results showing that more anthocyanin-related repressors with non-activity status in red-skinned *A. arguta* might be activated in green-skinned *A. arguta* in chromosome 16 (Supplementary Data Fig. S4b), which further led us to focus on *Aar11780*.

**Table 1:** Statistics of structural variations.

Type	TYH vs LC2 subA	TYH vs LC2 subB	TYH vs LC2 subC	TYH vs LC2 subD
Inversion	267	277	245	218
Translocation	6720	6814	6898	6763
CNV	17 271	17 010	15 779	15 791
PV	37 944	37 173	35 286	35 072
AV	50 657	50 182	48 914	47 953
PAV	88 601	87 355	84 200	83 025

### *AaCBP60B-like* responds to exogenous calcium chloride treatments

**Figure 3:**
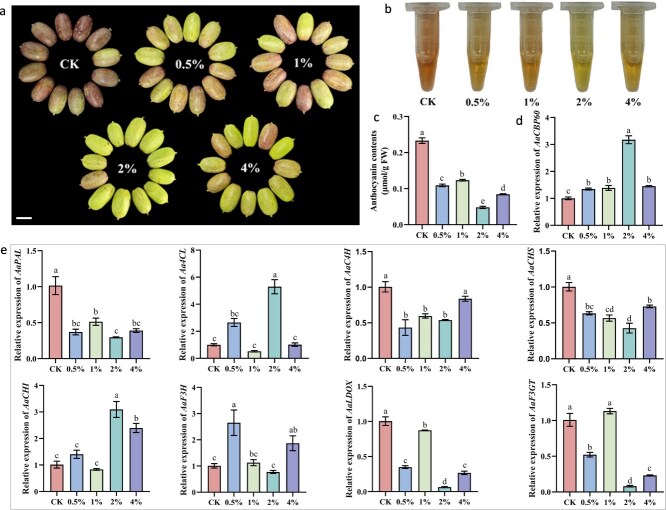
*AaCBP60B-like* response to exogenous Cacl_2_ treatment regulating anthocyanin biosynthesis. (a) Effect on fruit skin color of exogenous CaCl_2_ treatments with concentration gradient. Scale bar = 1 cm. (b) Anthocyanin extracts of fruit skin samples with concentration gradient of CaCl_2_. (c) Anthocyanin content of fruit skin samples with concentration gradient of CaCl_2_. Different lowercase letters represent significant differences at *P* < 0.05 level. (d) Relative expression of *AaCBP60B-like* in fruit skin samples with concentration gradient of CaCl_2_. (e) Relative expression of anthocyanin biosynthesis genes in fruit skin samples with concentration gradient of CaCl_2_. Different lowercase letters represent significant differences at *P* < 0.05 level.

We cloned *Aar11780* from a green-skinned sample with a full cDNA length of 1917 bp encoding 638 amino acids, and performed BLAST analysis in NCBI protein alignment as well as conserved amino acid domain analysis, confirming *Aar11780* as a *CBP60B-like* gene, so we designated *Aar11780* as *AaCBP60B-like*. Next, we designed an exogenous calcium chloride treatment with a gradient of four concentrations, 0.5, 1, 2, and 4%, in red-skinned fruits. Compared with the control fruits without exogenous calcium chloride, the red color of four treated fruits was suppressed, the 2% treatment exhibiting the strongest inhibition (Fig. 3a). Anthocyanin extracts and content were lower in treated samples than in control (Fig. 3b and c), consistent with fruit color phenotype. Expression level of *AaCBP60B-like* was higher in treated samples than in control (Fig. 3d). Structural genes, especially late biosynthesis genes (*LBG*s) including *AaF3H*, *AaLDOX*, and *AaF3GT* involved in anthocyanin biosynthesis, were weakly expressed after exogenous calcium chloride treatments (Fig. 3e). Therefore, *AaCBP60B-like* responds to exogenous calcium chloride treatments and thus represses anthocyanin biosynthesis.

### *AaCBP60B-like* negatively regulates anthocyanin biosynthesis in *A. arguta* fruit skin

**Figure 4:**
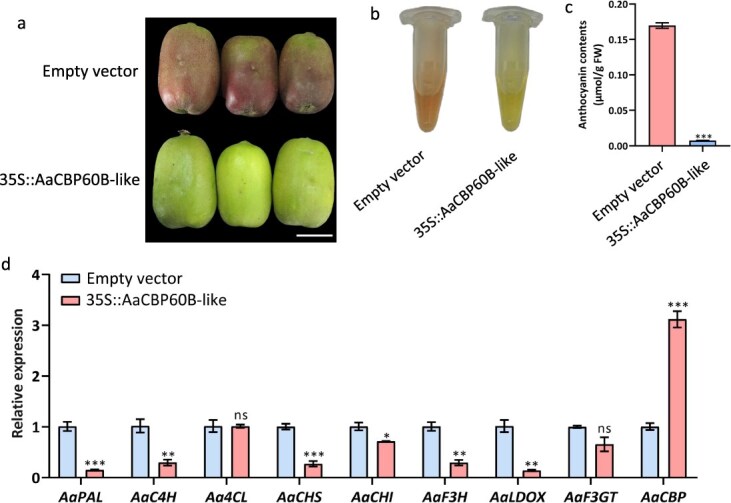
Overexpression of *AaCBP60B-like* in *A. arguta* fruits. (a–c) Color phenotype, anthocyanin extracts, and anthocyanin content of fruits injected with 35S-*AaCBP60B-like* or empty vector. (d) Relative expression of anthocyanin biosynthetic genes and *AaCBP60B-like* in fruits injected with 35S-*AaCBP60B-like* or empty vector. Values are means ± standard deviation for three replicates. Statistical significance: ^*^P < 0.05, ^**^P < 0.01, ^***^P < 0.001, ns, not significant.

To investigate the function of *AaCBP60B-like*, we constructed overexpressing and silencing recombinants for transient injections. Overexpression of *AaCBP60B-like* in red-skinned fruits resulted in little accumulation of red pigments compared with fruits injected with empty vector, which showed normal accumulation of pigments (Fig. 4a). Anthocyanin extracts were consistent with the color phenotype (Fig. 4b). Anthocyanin content was significantly lower in overexpressing samples than in the control (Fig. 4c), as was the relative expression of anthocyanin-related genes, in particular *LBG*s (Fig. 4d). On the contrary, expression-silencing fruits injected with TRV1/TRV2 + *AaCBP60B-like* significantly accumulated red pigments in fruit skin, but this did not occur in fruits injected with TRV1/TRV2 as the control (Supplementary Data Fig. S10a). Anthocyanin extracts and content were higher in silencing samples than in the control (Supplementary Data Fig. S10b and c), as was the expression of anthocyanin biosynthetic genes (Supplementary Data Fig. S10d). Overexpression and silencing assays both proved that differential expression of *AaCBP60B-like* induces two obvious phenotype differences in fruit color in *A. arguta*.

### Promoter activity of *AaCBP60B-like* differs in red- and green-skinned *A. arguta*

**Figure 5:**
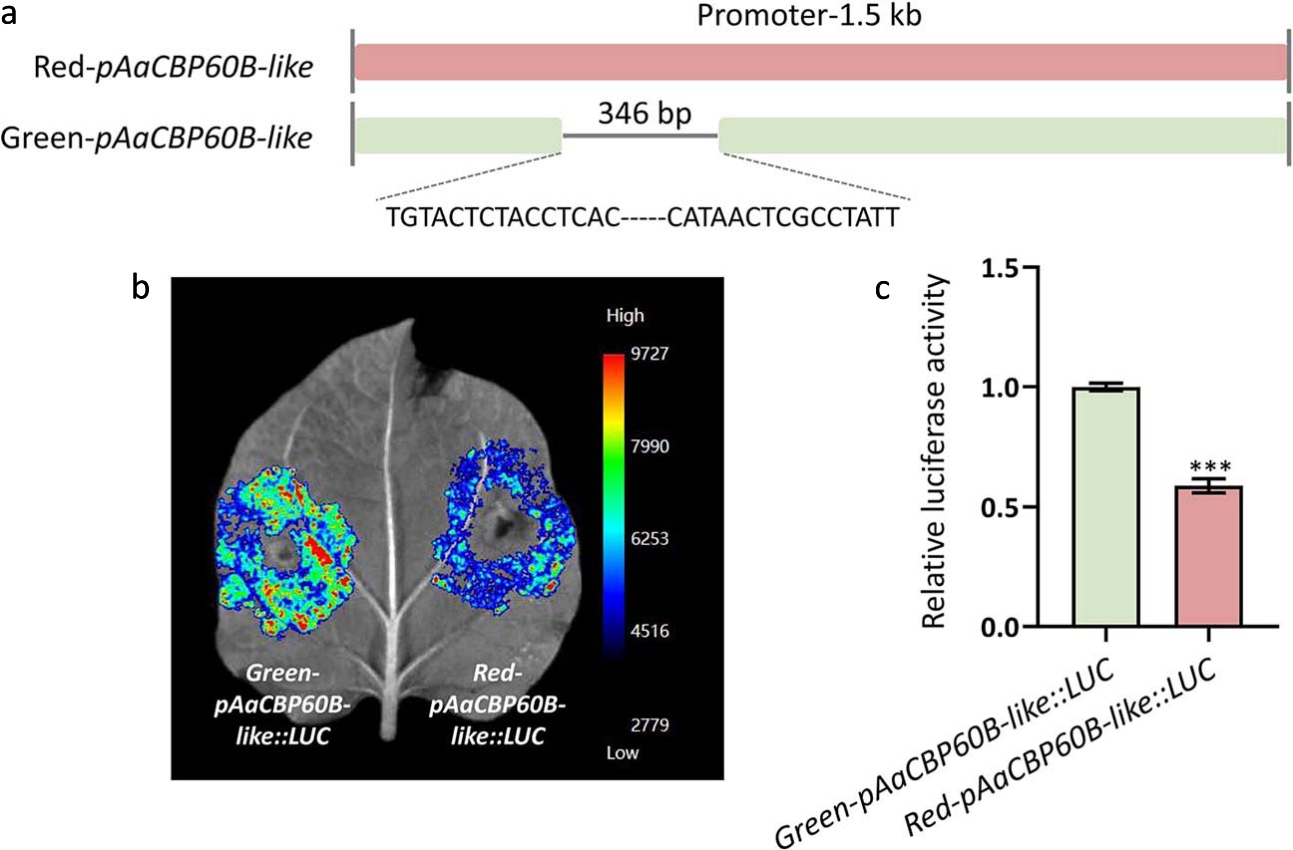
Activity analysis of *AaCBP60B-like* promoter with 346-bp InDel variation. (a) Promoter diagram of *AaCBP60B-like* with 346-bp InDel in red and green *A. arguta*. (b) Activities of *AaCBP60B-like* promoter with 346-bp insertion and deletion were analyzed by the luminescence intensity imaging system. Blue and red represent low and high luminescence intensity, respectively; c Relative luciferase activity of *AaCBP60B-like* promoter with 346-bp insertion and deletion. Values are means ± standard deviation for three replicates. Statistical significance: ^***^P < 0.001.

The expression of *AaCBP60B-like* exhibited a significant difference between red- and green- skinned *A. arguta*, and gene expression generally derived from either upstream or downstream regulation. The structure variation of 2 Mb window upstream and downstream of *AaCBP60B-like* between three *A. arguta* genomes, ‘Tianyuanhong’, LC2 [27], and M1 [29], showed that *AaCBP60B-like* is a conserved gene among the three genomes (Supplementary Data Fig. S11). Considering LC2 as a green-skinned cultivar, we analyzed structural variations of chromosome 16 between the ‘Tianyuanhong’ and LC2 genomes, and found translocation, inversion, and duplication showed a ordinary distribution (Supplementary Data Fig. S12). So we believed the differential expression of *AaCBP60B-like* between red- and green- skinned *A. arguta* probably resulted from structural variations. We randomly selected three red-skinned *A. arguta* cultivars, ‘Tianyuanhong’, RB-4, and ZHB, and three green-skinned *A. arguta* cultivars, LC2, ‘Changjiang-1’, and ZLB, to clone ~1.5 kb upstream of *AaCBP60B-like* ATG, and found that a 346-bp InDel is specifically identified in red-skinned and green-skinned *A. arguta* cultivars. This typical variation between the ‘Tianyuanhong’ and LC2 genomes was additionally confirmed by PCR and Sanger sequencing (Supplementary Data Fig. S13). Furthermore, we randomly selected 12 red-skinned and 12 green-skinned *A. arguta* accessions to check the conservation of variation. This showed that this 346-bp InDel could be used as a marker; specifically, the single band suggests a homozygous variation in red-skinned accessions, whereas two bands indicate heterozygosity in green-skinned accessions (Supplementary Data Fig. S14). Besides this InDel, several SNPs were also commonly distributed in the promoter of *AaCBP60B-like*. To cover the SNPs from different genotypes as much as possible, we selected the promoter sequence of RB-4 and ‘Changjiang-1’ (Supplementary Data Fig. S15), containing the most SNPs among these genotypes, to construct a vector for use in the LUC (luminescence intensity imaging system) assay. The solitary InDel is a 346-bp deletion in ‘Changjiang-1’, while there is a normal promoter in RB-4 (Fig. 5a). To explore effect of this 346-bp InDel on promoter activity, we conducted LUC assays in tobacco leaves by transient injection. As expected, the promoter with the 346-bp deletion had higher activity than that without variation in ‘RB-4’ both in luminescence intensity (Fig. 5b) and relative luciferase activity (Fig. 5c). A significant difference in *AaCBP60B-like* expression was detected between RB-4 and ‘Changjiang-1’ (Supplementary Data Fig. S16), which possibly derived from this InDel variation. Furthermore, we investigated correlation between *AaCBP60B-like* and anthocyanin synthesis genes from RNA-seq data and found *AaPAL*, encoding phenylalanine ammonia lyase as the first step of flavonoid pathway, had the highest correlation with *AaCBP60B-like* (Supplementary Data Fig. S17, Supplementary Data Table S13).

## Discussion

Gene accuracy expression and regulation and the interaction between regulation elements occur in the 3D architecture formed by chromosome interfolding, which plays a crucial role in biological phenotype formation. Hi-C, an innovative technology for studying the interaction of neighbor chromatin, is frequently applied in various species [13, 30]. However, Hi-C has barely been utilized in fruit trees so far. In the current study we performed Hi-C experiments with two biological replicates from red and green fruit skin of *A. arguta*. Chromatin interactions showed large Pearson correlations, which suggested high reproducibility of the Hi-C assays as well as the reliability of the Hi-C data (Supplementary Data Fig. S1). A and B compartments examined by PCA showed more euchromatin in A and centromeres and pericentromeric mainly in B, which is consistent with the basic biological specificity of long-range compartmentalization [26]. By further analysis of A and B compartment distribution throughout the genome, we found, in chromosome 7 and chromosome 16, that B compartments (containing more silencing genes) in red skin converted to A compartments (containing more active genes) in green skin (Fig. 1, Supplementary Data Fig. S4), implying some anthocyanin-related silencers in red skin might be converted into activators in green skin in these two chromosomes. The A/B-compartment transition is a common rule in plants. Sometimes changes in external environmental conditions would also result in the A/B-compartment transition. Eigenvalue analysis suggested the first and second B compartments under the normal condition converted into A compartments under heat stress to form a larger A compartment in chromosome 6 in the rice genome [23], indicating that the genes in chromosome 6 with a high possibility of response to heat stress provide a more precise condition or focus for candidate gene screening. Here, the candidate negative regulators involved in anthocyanin biosynthesis we are looking for are most likely located on chromosome 7 and chromosome 16. To cover the candidate genes as much as possible, we set a 3-Mb region upstream and downstream of the A/B compartment transition in chromosome 7 and chromosome 16, and a total of 249 genes were extracted for a robust bioinformatics approach by constructing a four-way Venn diagram, as well as a WGCNA based on the RNA-seq data. Finally, we focused on *AaCBP60B-like*, which had a negative correlation with fruit coloration. Calmodulin-binding protein 60 (*CBP60*)-like proteins are atypical transcription factors in plants [31]. There is a total of eight *CBP60* members in the *Arabidopsis thaliana* genome, and most of them mediate immune gene expression for disease resistance [32, 33]. An exception is that *AtCBP60g* functions as a negative regulator in *Arabidopsis* anthocyanin accumulation induced by drought, sucrose, and kinetin [28], which is line with the negative role of *AaCBP60B-like* in anthocyanin biosynthesis of *A. arguta* based on overexpression and silencing assays (Fig. 4; Supplementary Data Fig. S10), suggesting the potential role of *AaCBP60B-like* in immune resistance by anthocyanin mediation in *A. arguta*, which will be focused on or explored in our further study.

**Figure 6:**
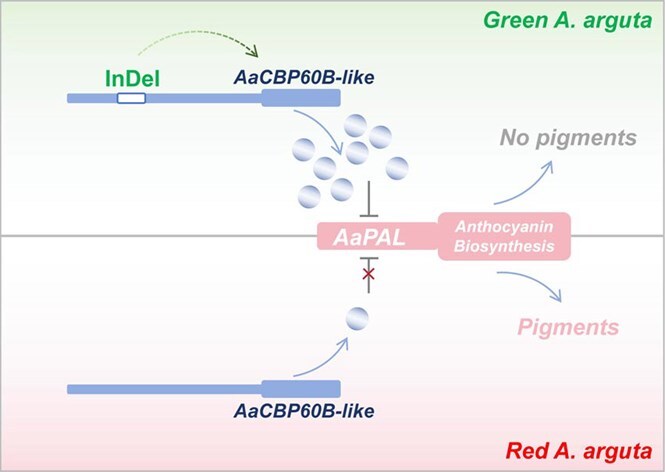
Proposed model of *AaCBP60B-like*-mediated anthocyanin regulation in different colored *A. arguta*. In green *A. arguta* the InDel variation induces high activity, resulting in high expression of *AaCBP60B-like*, thus more protein is produced to suppress anthocyanin biosynthesis. In red *A. arguta* there is less AaCBP60B-like production because of the absence of the InDel in the promoter, so there is not enough to repress anthocyanin biosynthesis and thus normal pigments accumulate.

*AaCBP60B-like* was differentially expressed in red- and green-skinned *A. arguta*, which usually results from the promoter regulation. We cloned a 1.5-kb length of promoter sequences from red- and green-skinned *A. argut*a, and found a 346-bp InDel variation located ~1 kb upstream of ATG resulting in differential promoter activity (Fig. 5). Further expression and correlation analysis revealed that *AaCBP60B-like* negatively regulates anthocyanin biosynthesis possibly by targeting *AaPAL* (Supplementary Data Fig. S17). Based on the results in this study, we propose a simple model of *AaCBP60B-like*-mediated anthocyanin regulation in *A. arguta* (Fig. 6). A deletion in the promoter induces high expression of *AaCBP60B-like* to produce more proteins to negatively regulate anthocyanin biosynthesis in green-skinned *A. arguta*, while an insertion in the promoter induces a low expression level of *AaCBP60B-like* to produce few proteins, not enough to negatively regulate anthocyanin biosynthesis in red-skinned *A. arguta*. Furthermore, our analysis of 32 *CBP60B*-homologous genes from various *Actinidia* species revealed that *Aar11780* clusters specifically with its *A. arguta* homolog *Aarlcv1a16g222130* (Supplementary Data Fig. S18), indicating that this 346-bp InDel-mediated regulatory mechanism is unique to *A. arguta* and absent in related species, consistent with the species-specific anthocyanin regulation we observed. All in all, we found a new negative regulator of anthocyanin biosynthesis in *A. arguta*, though new issues, such as which of the upstream regulators targets *AaCBP60B-like*, and the specific mechanism of the effects of the InDel on expression differences of *AaCBP60B-like*, need to be further explored using DAP-seq/Chip-seq and Y1H library constructs using promoters with InDel variation. Moreover, the coloration of the skin, as the outermost layer of the fruit, is highly susceptible to environmental factors such as light. For instance, visible light has been shown to induce *PpHYH* transcription, thereby regulating anthocyanin accumulation and peach skin pigmentation [34]. A key focus of our follow-up research will be to investigate whether light similarly modulates *A. arguta* fruit skin coloration by mediating *AaCBP60B-like* expression. This will help elucidate the molecular mechanisms underlying color formation in response to external environmental cues.

### Conclusions

The construction of the Hi-C differential landscape of red- and green-skinned *A. arguta* reveals that A/B compartment transition, in particular in chromosome 7 and chromosome 16, plays a crucial role in fruit skin coloration, and that *AaCBP60B-like* is the candidate gene, the function of which was confirmed by exogenous calcium chloride treatments and overexpression/silencing assays showing negative regulation of anthocyanin biosynthesis. A 346-bp InDel variation located in the *AaCBP60B-like* promoter resulted in activity differences in red- and green-skinned *A. arguta*. Our study not only provides the first 3D chromosome organization but also a novel negative regulator of anthocyanin regulation in red-skinned and green-skinned *A. arguta*.

## Materials and methods

### Plant materials

Two *A. arguta* cultivars, ‘Zhonghongbei’ (ZHB, red-skinned) and ‘Zhonglvbei’ (ZLB, green-skinned), grown at Xinxiang Experimental Station of Zhengzhou Fruit Research Institute in Henan Province, were selected as experimental materials for high-throughput chromosome conformation capture (Hi-C). Fruit peels from ZHB and ZLB (which remains green throughout development) were collected at three coloration stages: S1, 70 days after full bloom (ZHB is green); S2, 105 days after full bloom (ZHB transitions from green to red); and S3, 130 days after full bloom (ZHB is fully red). These samples were then used for RNA-seq analysis.

### Hi-C library construction and Illumina PE150 sequencing analysis

Raw sequencing reads were first filtered to generate high-quality clean data for downstream analysis. The filtering steps included adapter removal, trimming of bases with quality scores <3 at both ends, exclusion of bases with average quality scores <20, and removal of reads shorter than 50 bp. The Hi-C contact matrices (100-kb resolution) were normalized using the Lowess *z*-score method. A differential matrix was then generated by subtracting the normalized matrix of the control sample (ZLB) from that of ZHB, which was visualized as a heat map. Genomic compartments were classified into A compartments (typically transcriptionally active) and B compartments (generally inactive) based on the PCA-derived PC1 values and C-scores at 100-kb resolution [13, 35]. After assigning compartments to each bin in ZHB and ZLB, we compared the compartment states between the two samples, identifying four transition patterns: A-to-A (A2A), A-to-B (A2B), B-to-A (B2A), and B-to-B (B2B). TAD boundaries were determined using insulation score analysis [36–38]. TAD boundaries of ZHB and ZLB were compared, yielding unique boundaries for each sample and shared boundaries, which were visualized in a Venn diagram. Additionally, boundary aggregate analysis was performed using Coolpup.py [39] to assess the strength and conservation of unique and common boundaries. Chromatin loops were identified at 5-, 10-, and 20-kb resolutions, and the combined results were used to analyze structural differences between the red- and green-skinned samples [40, 41]. We further compared *cis*- and *trans*-significant interactions between ZHB and ZLB, applying *P*-value and *Q*-value thresholds of 0.01 to assess differential chromatin contacts.

### Variation identification by comparative genomics analysis

We used BLASTp (E-value cutoff of 1 × e^−5^) for homologous sequence alignment. OrthoFinder (v2.3.12) was employed to analyze the BLAST results, cluster gene families, and identify core and dispensable genes [42]. To identify genomic variations between the ‘Tianyuanhong’ and LC2 genome, we performed structural variant calling using SyRI [43], which detected SNPs, InDels, structural variations (inversion and translocation), CNAs, and PAVs. Variant annotation was conducted using ANNOVAR [44].

### Venn diagram for candidate gene screening

We performed time-course DEG analysis between the red-skinned cultivar ZHB and the green-skinned cultivar ZLB (as control), and constructed a four-way Venn diagram, using TB-tools [45].

### ATAC-seq analysis

ATAC-seq was performed using a modified sucrose precipitation protocol [46]. Nuclei were isolated from fruit skin samples of ZHB and ZLB, followed by Tn5 transposase treatment (37°C for 30 min). After DNA purification, libraries were prepared by PCR amplification and sequenced on the Illumina PE150 platform (paired-end 150 bp). Raw reads were filtered to obtain clean data, which were then aligned to the *A. arguta* ‘Tianyuanhong’ reference genome [24]. Read alignment processing was performed using Samtools [47], and biological replicates were merged into a single BAM file. Finally, BAM files were converted to BigWig format using bamCoverage from deepTools [48].

### Weight gene coexpression network analysis

We performed WGCNA using the R package WGCNA [49] on 249 significant DEGs identified across three developmental stages in the red-skinned *A. arguta* cultivar ZHB. The adjacency matrix was converted to a topological overlap matrix (TOM), and genes were clustered into modules based on TOM dissimilarity. Analysis parameters included a soft thresholding power of 7 and a minimum module size of 15. Modules showing high similarity (*r* > 0.75) were merged. Gene module–phenotype correlations were computed using default parameters. Genes with high module membership (|MM| > 0.8) were identified as hub genes likely possessing important biological functions.

### Exogenous calcium chloride treatments

Fruits from the red-skinned cultivar ZHB were treated with exogenous calcium chloride at four concentration (0.5, 1, 2, and 4%). Phenotypic changes, particularly in fruit coloration, were documented photographically. Three days post-treatment, fruit skin samples were collected using a sterile scalpel, immediately flash-frozen in liquid nitrogen, and stored at −80°C for subsequent analysis.

### Transient overexpression in red-skinned *A. arguta* fruits

The full-length coding sequence (CDS) of *AaCBP60B-like* was amplified from ZLB using gene-specific primers containing homologous arms and subsequently cloned into the plant binary vector pBI121 to generate the 35S-*AaCBP60B*-like overexpression construct with kanamycin resistance. The recombinant plasmid was introduced into *Agrobacterium tumefaciens* strain GV3101. For transient overexpression in red-skinned *A. arguta* fruits, we followed the established protocol [3], using ~30 fruits per infiltration experiment with three biological replicates. Specific primers used in this experiment are shown in Supplementary Data Table S14.

### Virus-induced gene silencing in green-skinned *A. arguta* fruits

Virus-induced gene silencing (VIGS) of *AaCBP60B-like* in green-skinned *A. arguta* fruits was performed via *Agrobacterium*-mediated transient transformation, following an established protocol [3]. A specific exon sequence of *AaCBP60B-like* was cloned into pTRV2 vector for targeted gene silencing. Fruits infiltrated with pTRV2 + pTRV1 served as negative controls. For each experimental group, ~30 fruits were infiltrated, with three biological replicates performed. Specific primers used in this experiment are shown in Table S14.

### LUC assays

The ~1.5-kb promoter regions of *AaCBP60B-like* were cloned from the red-skinned cultivar RB-4 and the green-skinned cultivar ‘Changjing-1’, then inserted into the linearized pGreenII 0800-LUC double-reporter [50] vector to generate *Red-pAaCBP60Bpro::LUC* and *Green-pAaCBP60B-like::LUC* constructs. These recombinant vectors were transformed into *A. tumefaciens* strain GV3101 containing the pSoup helper plasmid. Bacterial suspensions were prepared in infiltration buffer (10 mM MES, 10 mM MgCl_2_, 150 mM AS). For transient expression assays, 4- to 5-week-old *Nicotiana benthamiana* leaves were infiltrated following previous descriptions [51, 52]. Promoter activity was analyzed 48–72 hours post-infiltration using both fluorescence imaging (LASER900, BIO-OI, Guangzhou, China) and the Dual-Luciferase^®^ Reporter Assay System (E1910; Promega). The experiments were performed with three replicates. Primers used in LUC are listed in Table S14.

### Anthocyanin measurement

Anthocyanin extraction and quantification were performed using a Plant Anthocyanin Content Assay Kit (Boxbio, Beijing, China) according to the manufacturer’s protocol. Absorbance measurements were taken at 530 and 700 nm. Anthocyanin content was calculated using the kit’s specified formula.

### Quantitative real-time PCR

Total RNA was extracted from all samples using the Quick RNA Isolation Kit (Huayueyang Biotechnology Co., Ltd, Beijing, China) following the manufacturer’s protocol. RNA quality was verified by 1% agarose gel electrophoresis, while concentration and purity were determined using a NanoDrop 2000 spectrophotometer (Thermo Fisher Scientific, MA, USA). First-strand cDNA was synthesized with ReverTra Ace qPCR RT Master Mix FSQ-201 (TOYOBO, Osaka, Japan). RT–qPCR was performed as previously described [3], with three biological replicates per sample. Gene expression levels were quantified using the 2^-ΔΔCt^ method [53]. Primers used in this assay are listed in Table S14.

### Statistical analysis

Statistically significant differences were assessed by Student’s *t*-test (*P <* 0.05). All analyses were conducted using GraphPad Prism 8 (GraphPad Software Inc., San Diego, CA, USA). MEGA 6.0 software and the online tool EvolView were used for phylogenetic tree construction. qRT–PCR and anthocyanin content data are presented as means ± standard error of three biological replicates.

## Supplementary Material

Web_Material_uhaf183

## Data Availability

All relevant data can be found within the article and its supplementary materials.
