# Supplementary material for: Combination of 3D chromatin architecture and omics analysis provides insight into anthocyanin regulation in Actinidia arguta
Source: Hortic Res. 2025 Jul 22;12(10):uhaf183. doi: 10.1093/hr/uhaf183 (PMC12539865; doi:10.1093/hr/uhaf183)
Supplement: Web_Material_uhaf183 [file web_material_uhaf183.zip › Supplementary Figures.pdf]

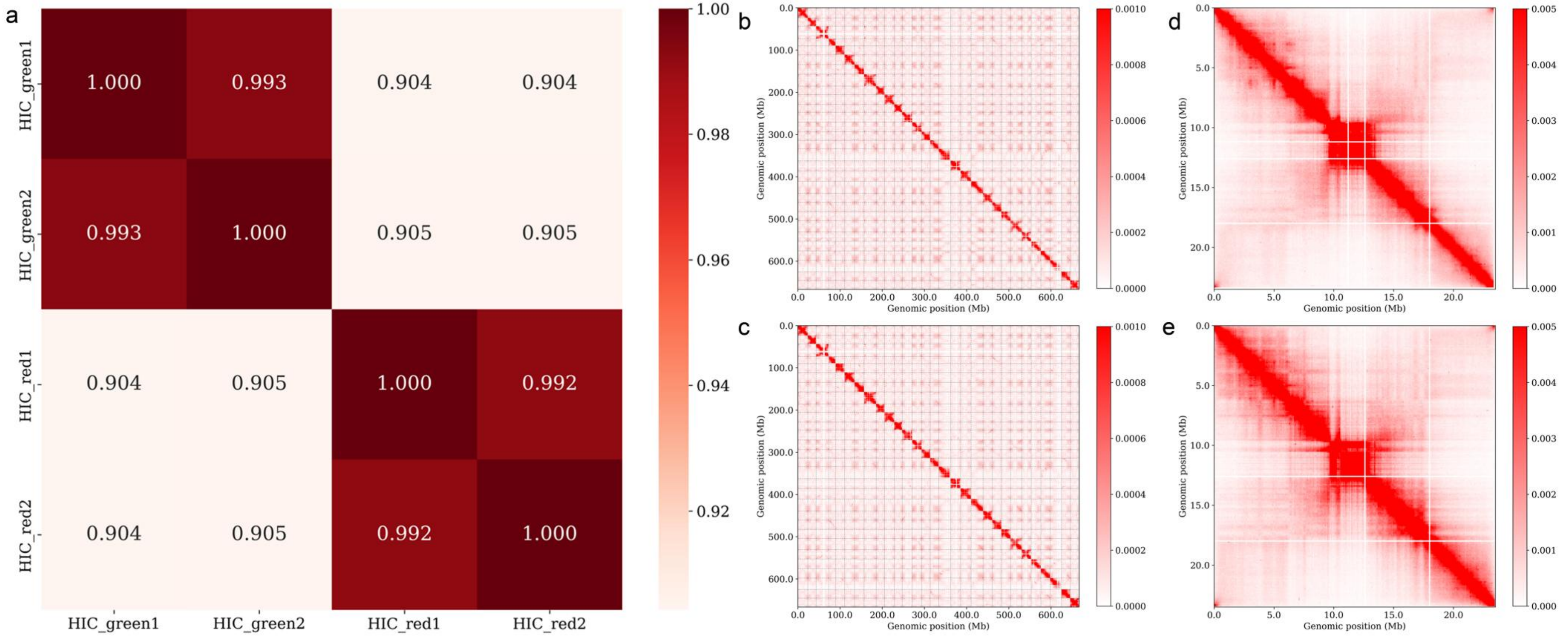

Fig. S1 Hi-C of red and green *A. arguta*. **a** Reproducibility of replicate experiments, including red-skinned and green-skinned *Actinidia arguta*. Deeper red color means stronger correlation between different replicates; **b** Hi-C interaction heatmap of whole-genome scale of 'ZHB'; **c** Hi-C interaction heatmap of whole-genome scale of 'ZLB'; **d** Hi-C interaction heatmap of randomly selected Chromosome 1 of 'ZHB'; **e** Hi-C interaction heatmap of randomly selected Chromosome 1 of 'ZLB'.

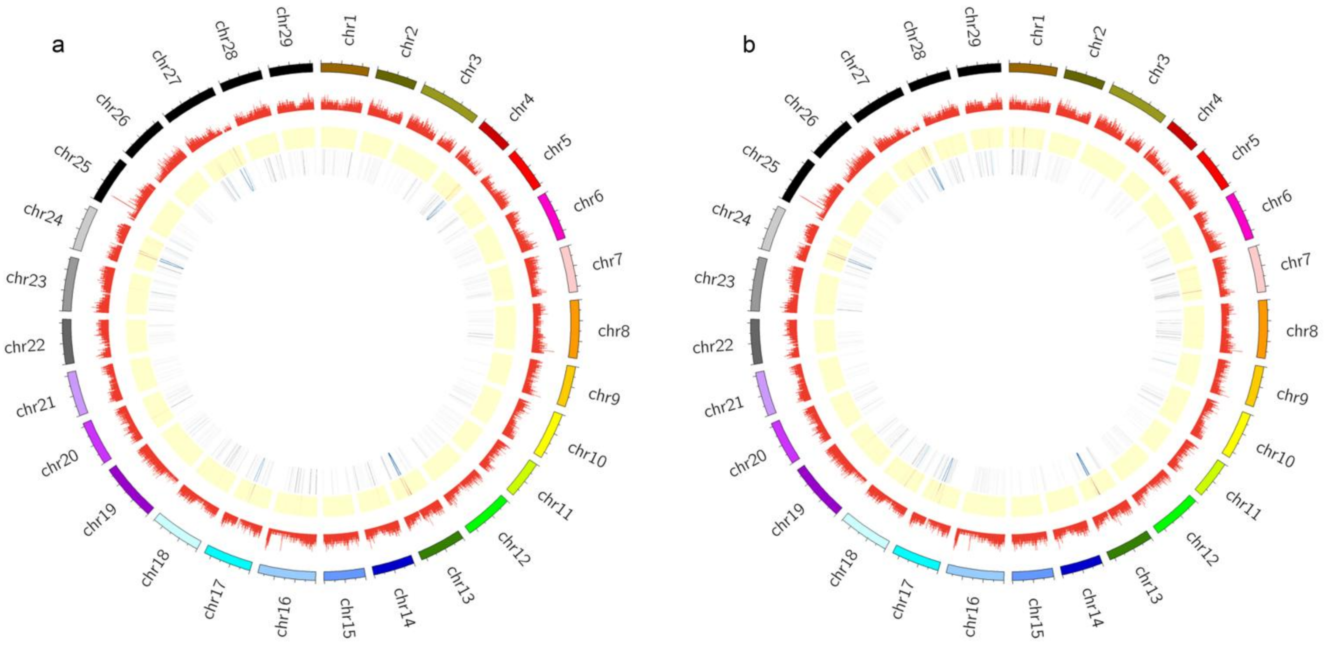

Fig. S2 Cis-interaction at whole genome level of red and green *A. arguta*. **a** Cis-interaction of red *A. arguta*; **b** Cis-interaction of green *A. arguta*.

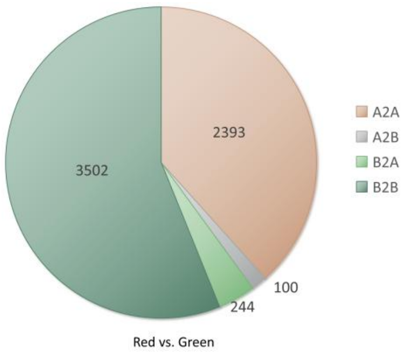

Fig. S3 Global compartment numbers divided by stable (A-A and B-B) and switching (A-B and B-A) compartments in red vs. green.

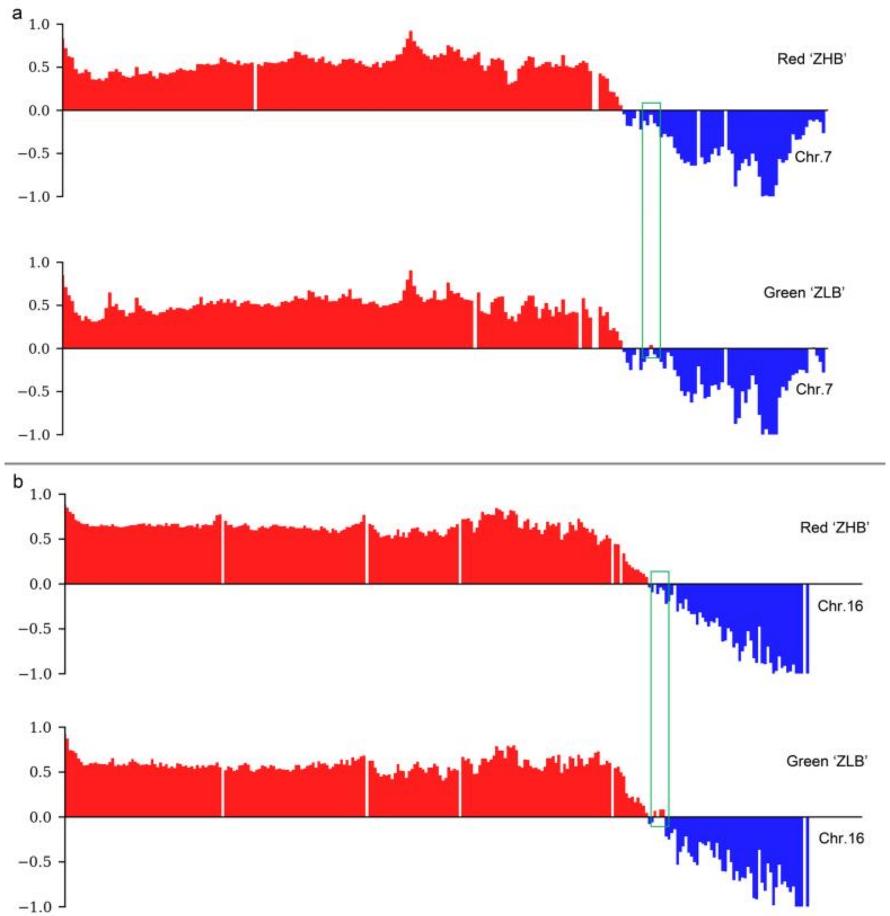

Fig. S4 PC1 scores for chromosomes of 'ZHB' and 'ZLB'. **a** PC1 scores for chromosome 7 of 'ZHB' and 'ZLB'; **b** PC1 scores for chromosome 16 of 'ZHB' and 'ZLB', green boxes highlight regions transitioning from B compartment in 'ZHB' to A compartment in 'ZLB .

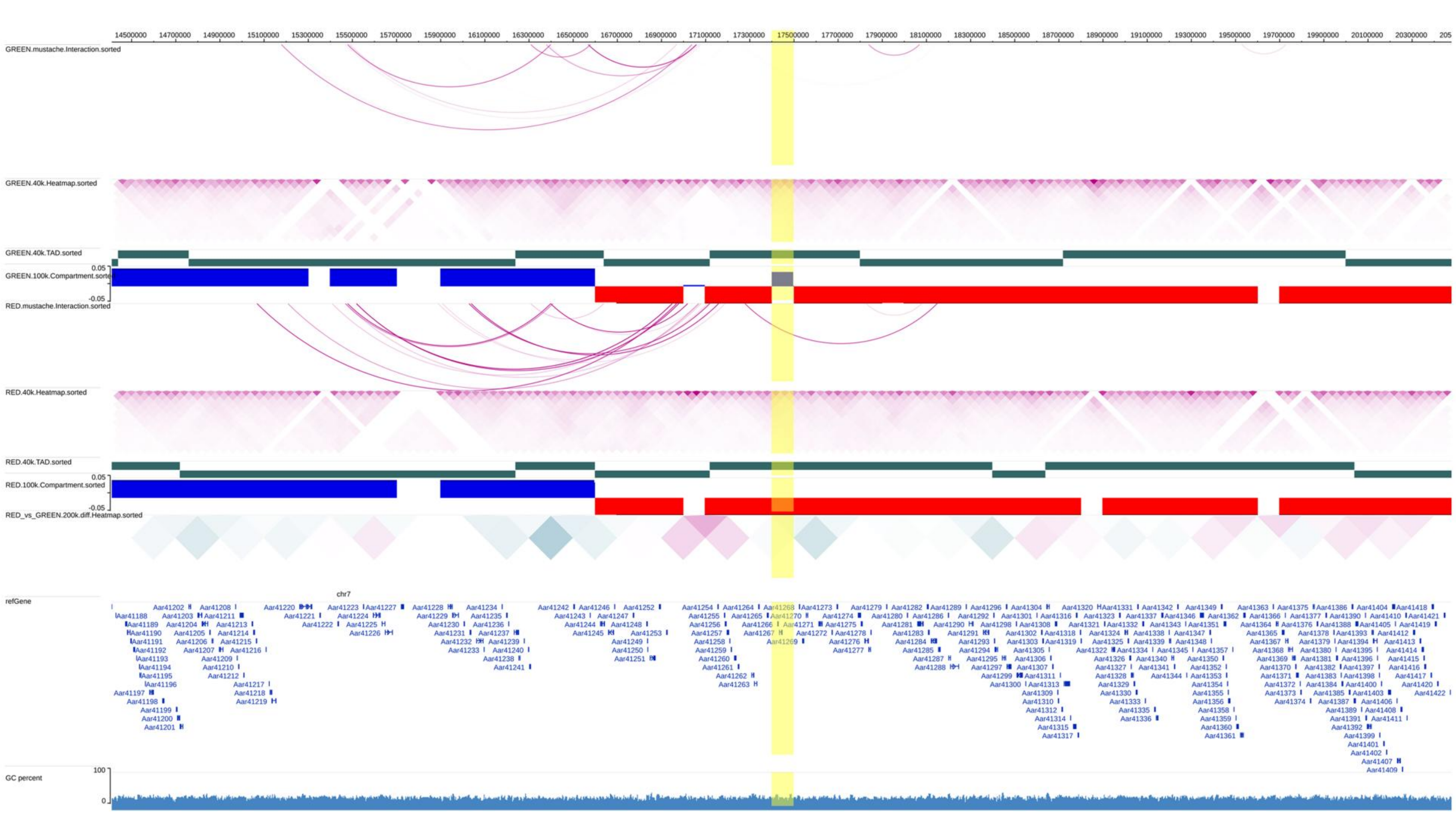

Fig. S5 Distribution of genes in the 3 Mb upstream and downstream of compartment transition in chromosome 7.

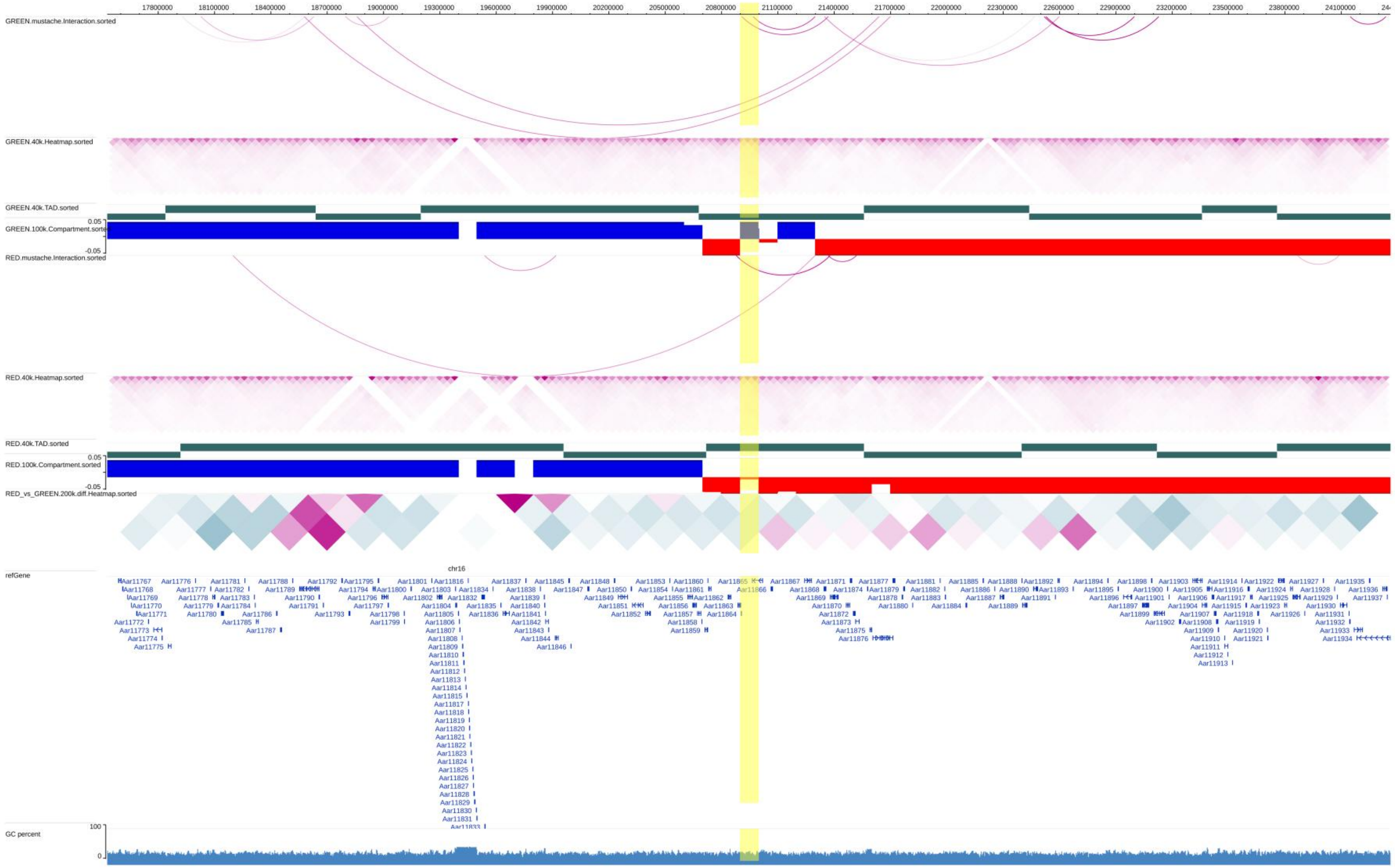

Fig. S6 Distribution of genes in the 3 Mb upstream and downstream of compartment transition in chromosome 16.

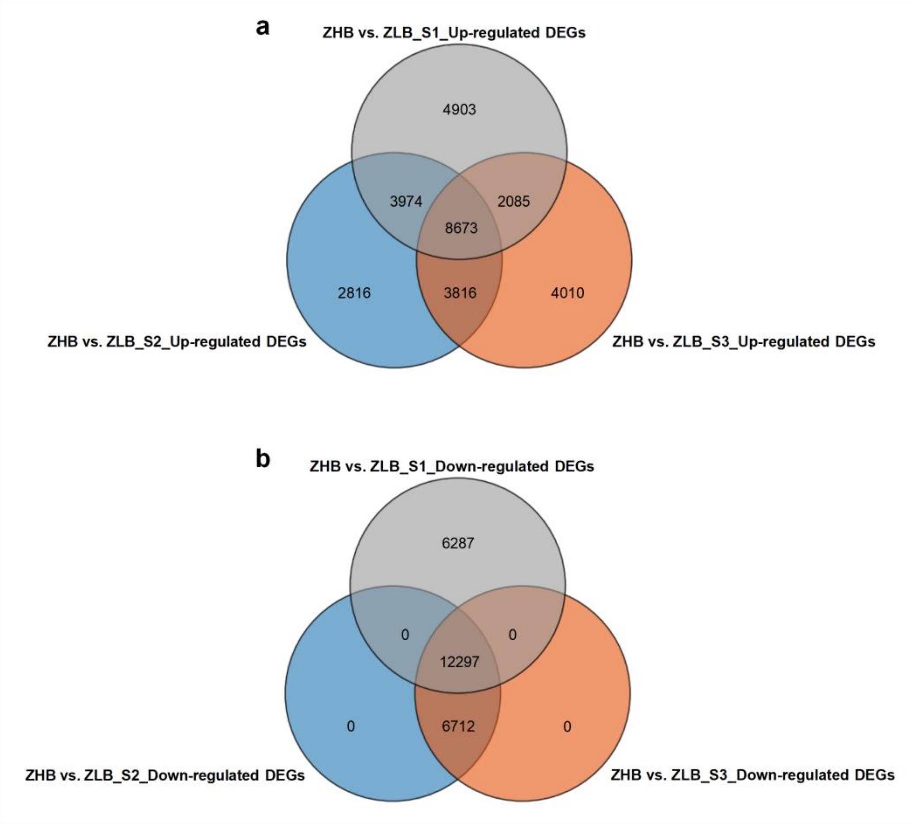

Fig. S7 A time-course (early, mid, and late-phase) differential gene expression (DEG) analysis comparing the red-skinned cultivar ZHB with the green-skinned cultivar ZLB (as control). a The up-regulated DEGs during the three comparisons ZHB vs. ZLB_S1, ZHB vs. ZLB_S2 and ZHB vs. ZLB_S3. b The down-regulated DEGs during the three comparisons ZHB vs. ZLB_S1, ZHB vs. ZLB_S2 and ZHB vs. ZLB_S3.

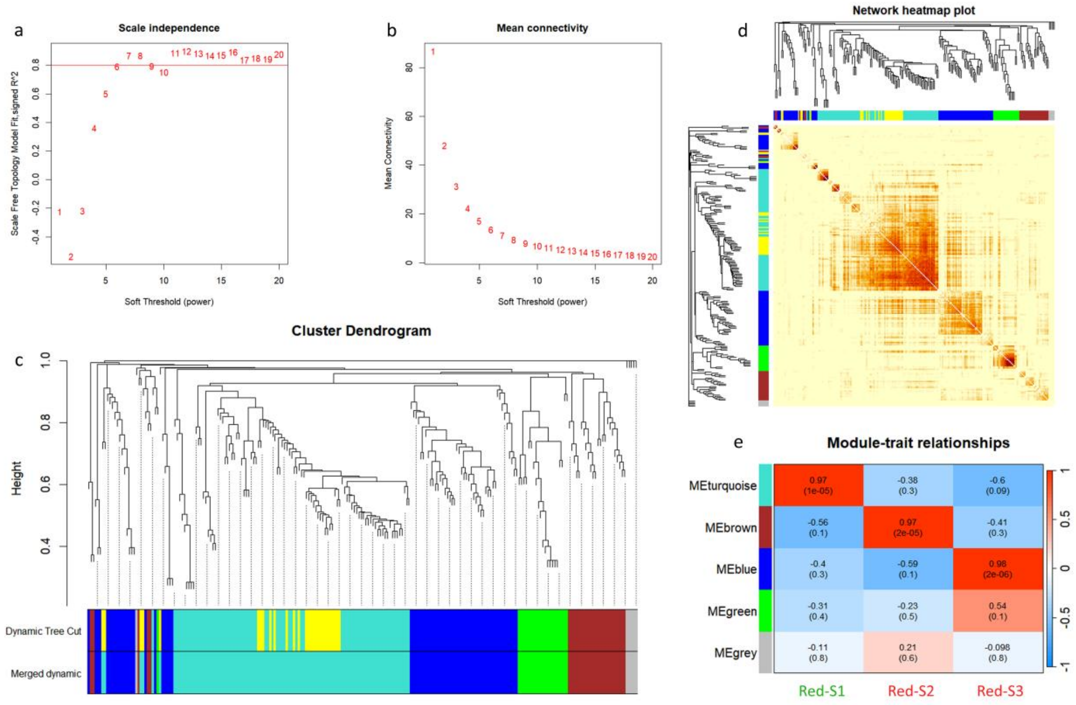

Fig. S8 WGCNA of genes in the upstream and downstream 3 Mb region in the compartment transition of Chr. 7 and Chr. 16. **a** The diagram of soft threshold and Scale Free Topology Model Fit, signed R^2; **b** The diagram of soft threshold and Mean Connectivity; **c** Hierarchical clustering tree illustrating 6 modules of co-expressed genes; **d** Heatmap plot of topological overlap in the gene network. Low topological overlap matrix (TOM) value is indicated by yellow color and higher TOM is indicated by progressively red color; **e** Heatmap of module-trait relationships. Each row represents a module indicated by different colors. The color key from blue to red represents correlation values from -1 to 1..

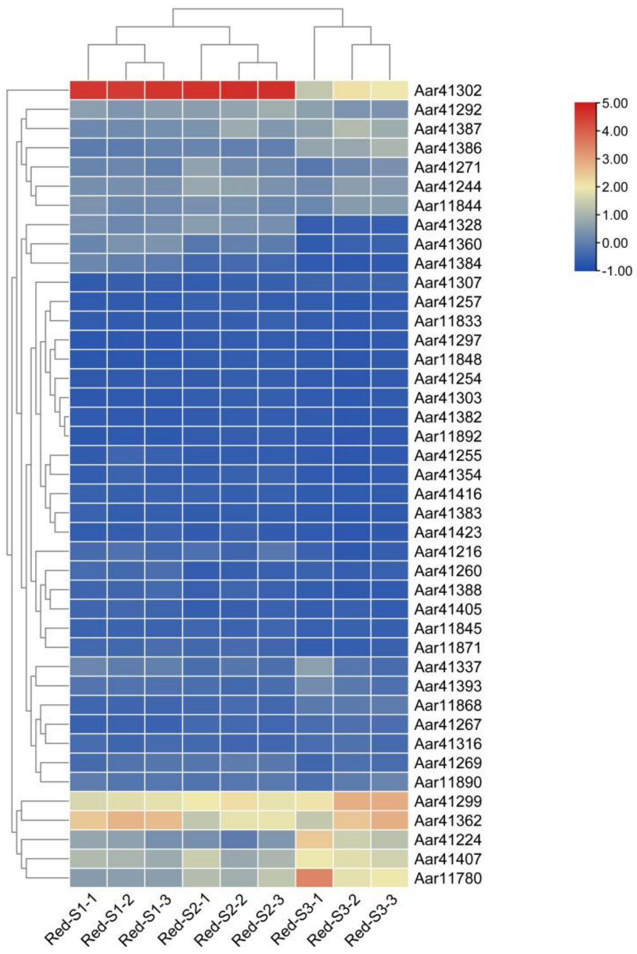

Fig. S9 Gene expression of 42 hub genes in different colored samples.

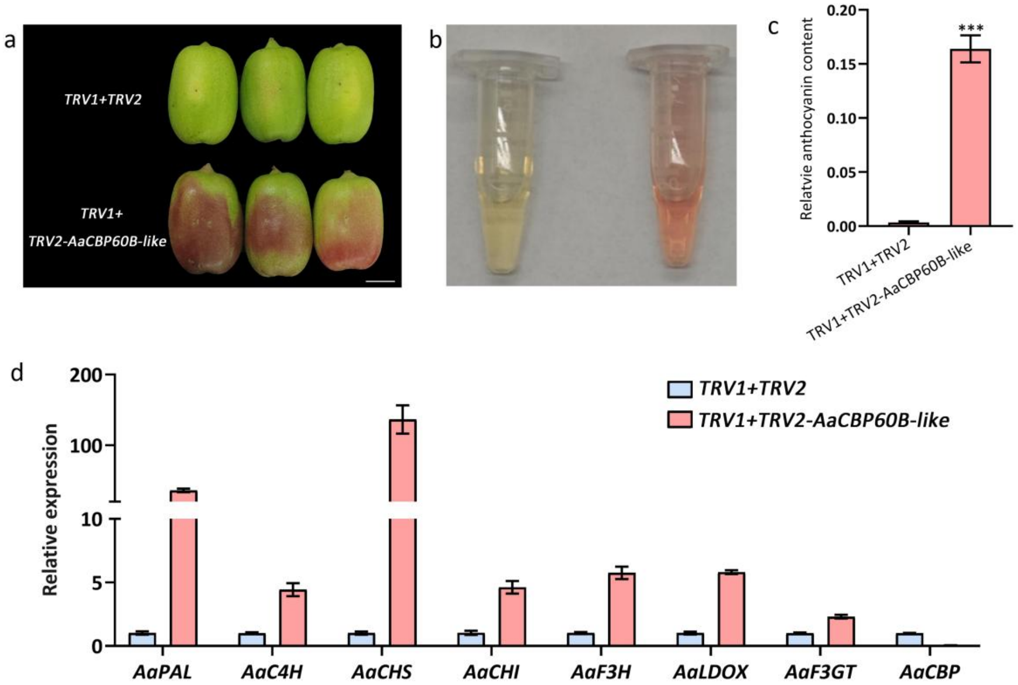

Fig. S10 VIGS-mediated transient silencing of AaCBP60B-like in *A. arguta* fruits. **a-c** Color phenotype, anthocyanin extracts, and anthocyanin content of fruits injected with TRV1/TRV2-AaCBP60B-like and TRV1/TRV2, respectively; **d** Relative expression of anthocyanin biosynthetic genes and AaCBP60B-like in fruits injected with TRV1/TRV2-AaCBP60B-like and TRV1/TRV2.

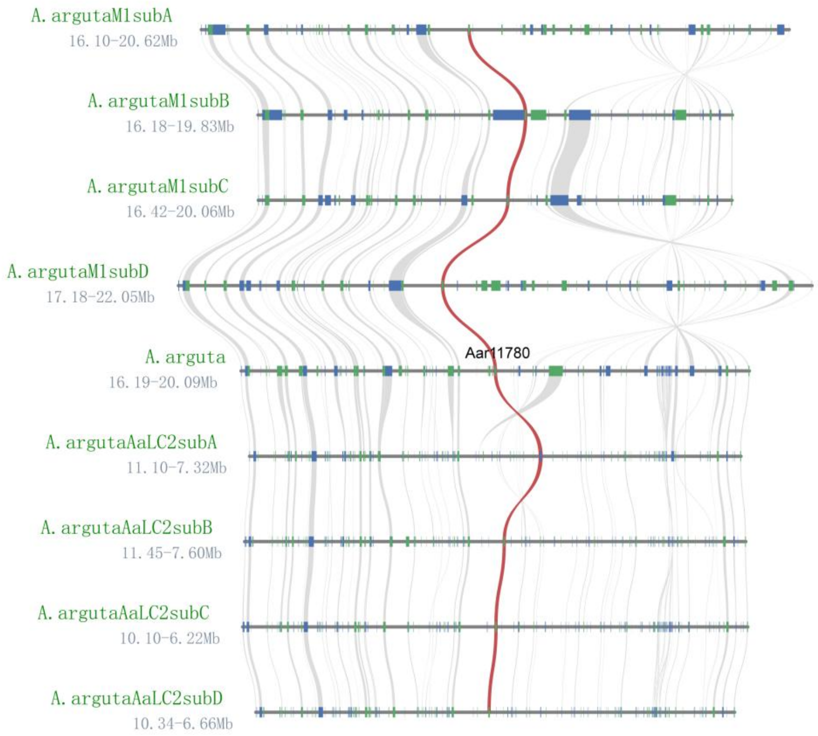

Fig. S11 Structure variation exhibition of 2 Mb region upstream and donwstream of Aar11780 among three *A. arguta* genome including 'Tianyuanhong' genome, 'LC2' genome and 'M1' genome.

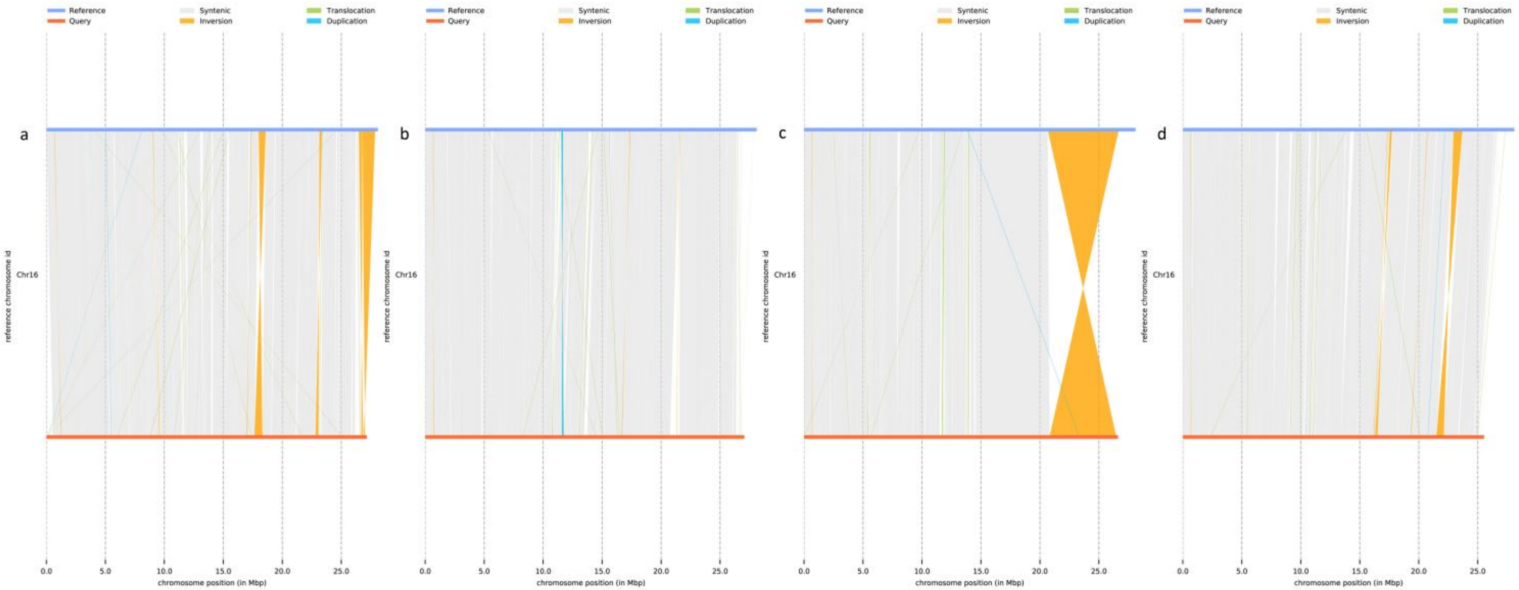

Fig. S12 Variation alignment of chromosome 16 between 'Tianyuanhong' genome and 'LC2' genome. **a**-**d** Chromosome 16 of 'Tianyuanhong' genome aligned to A-D haplotype of chromosome 16 of 'LC2' genome.

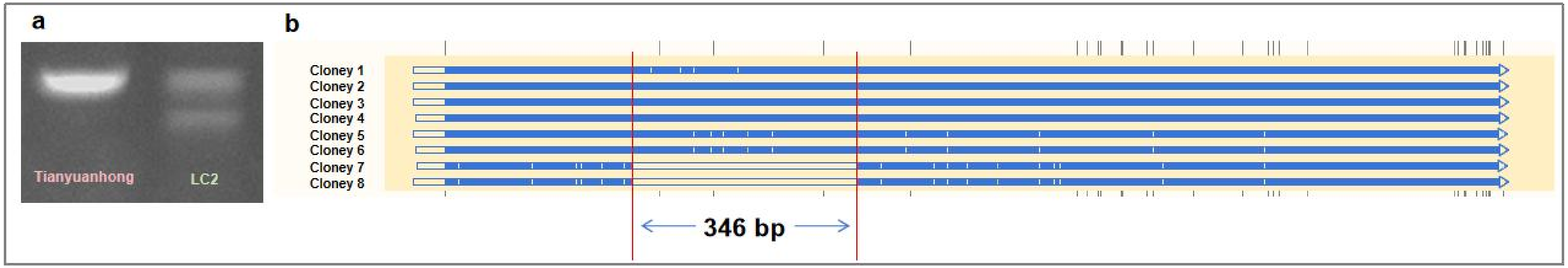

Fig. S13 Indel variation verification between 'Tianyuanhong' and 'LC2' genome. **a** PCR detection of Indel variation; **b** Specific sequence alignment of *AaCBP60B-like* promoter cloneys by Sanger sequencing.

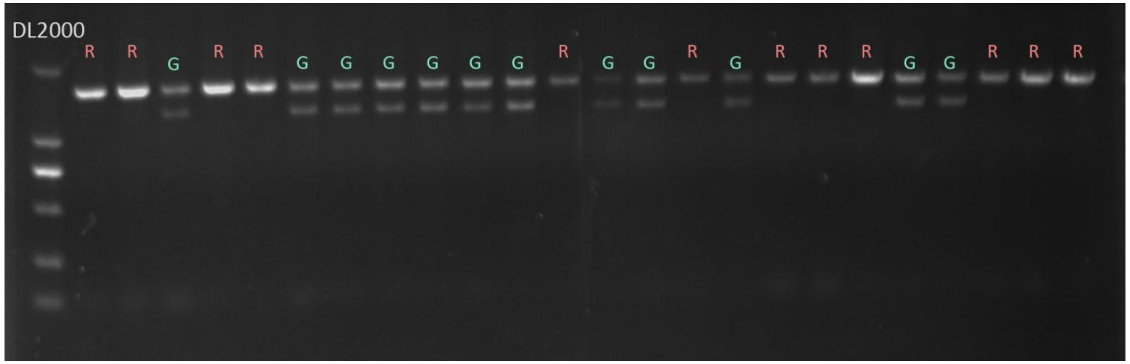

Fig. S14 Indel variation verification across 12 red-skinned and 12 green-skinned accessions.

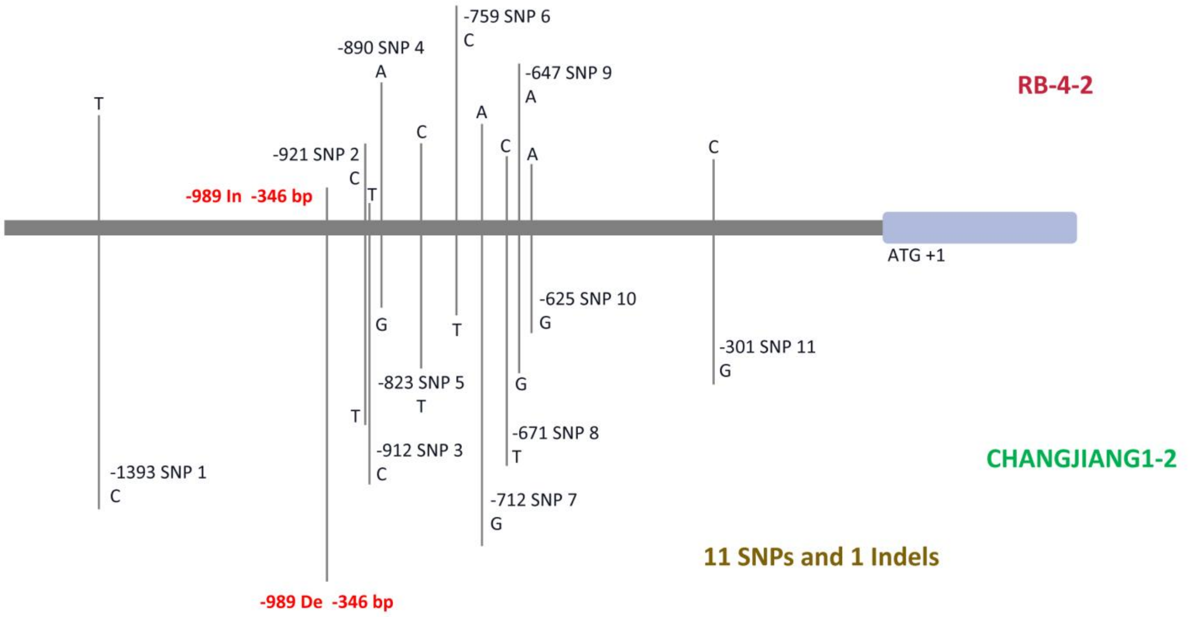

Fig. S15 Variation analysis of AaCBP60B-like promoter between red-skinned *A. arguta* 'RB-4' and green-skinned *A. arguta* 'Changjing-1'.

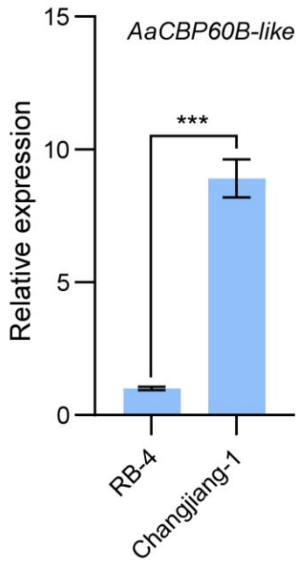

Fig. S16 The expression of *AaCBP60B-like* gene in red-skinned ‘RB-4’ and green-skinned ‘Changjiang-1’. Values are means ± SD for three replicates. Statistical significance: ***P < 0.001.

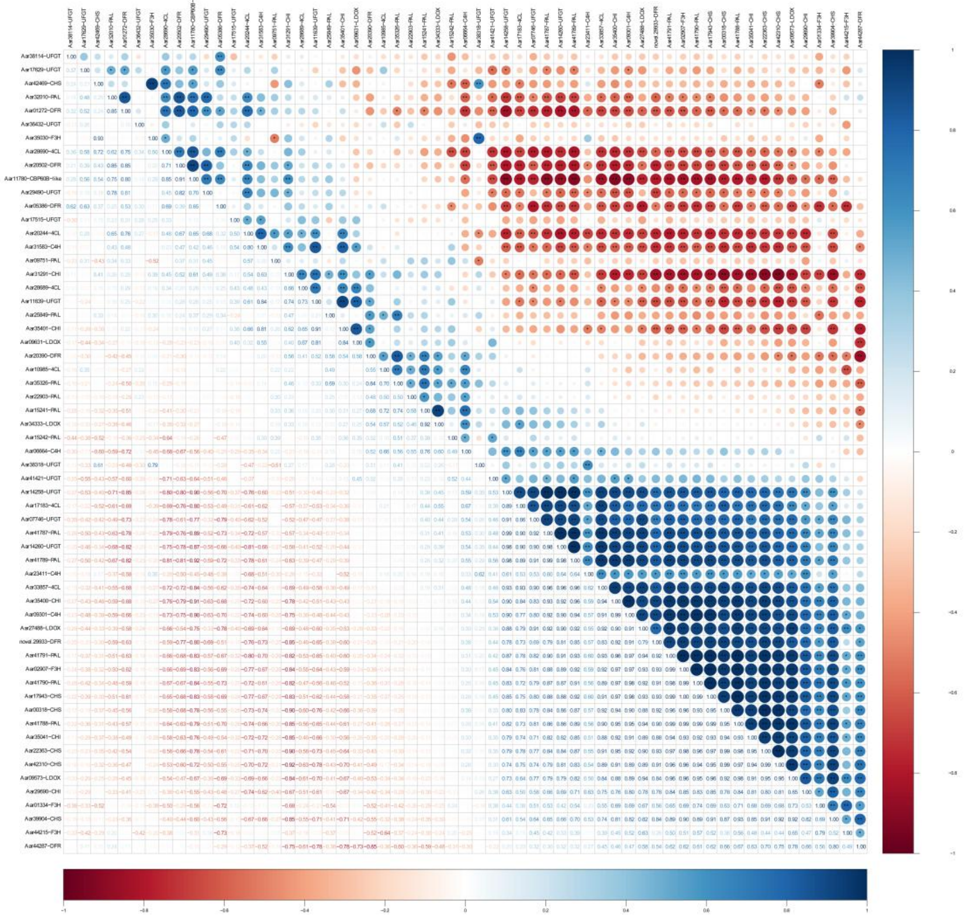

Fig. S17 The correlation analysis of gene expression between *AaCBP60B-like* and anthocyanin synthesis genes by R-package.

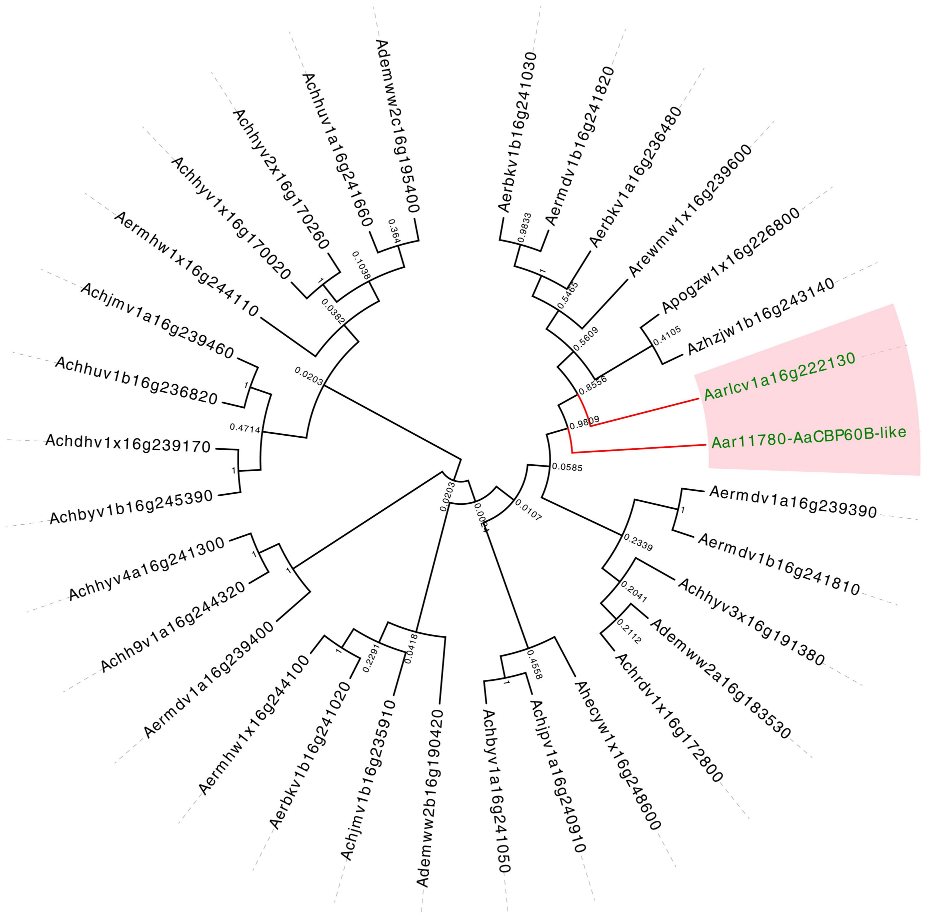

Fig. S18 The phylogenetic analysis of 32 CBP60B homologous genes from various *Actinidia* genus in the PanGenome data. The two *A. arguta CBP60B-like* gene clustered together.
